# Ablation of cystic thyroid nodules with or without the injection of ethanol prior to aspiration: a one-year follow-up study

**DOI:** 10.1007/s13304-026-02607-9

**Published:** 2026-05-05

**Authors:** Lorenzo Scappaticcio, Nicole Di Martino, Pamela Ferrazzano, Silvia Angelino, Michela Di Nuzzo, Miriam Longo, Alessandro Monaco, Giovanni Docimo, Maria Ida Maiorino, Katherine Esposito, Giuseppe Bellastella

**Affiliations:** 1 Department of Advanced Medical and Surgical Sciences, University of Campania “Luigi Vanvitelli”, Naples, 80138 Italy; 2 Unit of Endocrinology and Metabolic Diseases, AOU University of Campania “Luigi Vanvitelli”, Naples, 80138 Italy; 3 Department of Life Science, Health, and Health Professions, Link Campus University, Rome, 00165 Italy; 4 Unit of Thyroid Surgery, AOU University of Campania “Luigi Vanvitelli”, Naples, 80131 Italy

**Keywords:** Thyroid nodule, Ethanol ablation, Efficacy outcomes, Nodule volume

## Abstract

To evaluate the efficacy of ethanol ablation (EA) in treatment of cystic thyroid nodules (CTNs), according to procedure [with, contrariwise EA (contrEA), or without, classical (classEA), the injection of ethanol prior to aspiration] and injected ethanol amounts. We retrospectively reviewed consecutive patients treated with single-session EA for cytologically benign and symptomatic CTNs over four years. A total of 109 nodules were included [contrEA (*n* = 20) and classEA (*n* = 89)]. Twelve months after EA, median VRR was significantly higher in classEA group (93.1%) than contrEA (85.7%), while local symptoms did not significantly differ. Only the monolocular aspect of nodules could predict achievement of the three high efficacy outcomes (i.e., VRR > 75%, overall VAS score = 0, CS = 1), regardless of ethanol injected, EA procedure, nodule volume. EA, with or without the injection of ethanol prior to aspiration, is effective and positively influenced by the monolocular aspect of thyroid nodules, regardless of the EA procedure.

**Supplementary Information:**

The online version contains supplementary material available at https://doi.org/10.1007/s13304-026-02607-9.

## Introduction

Ultrasound (US)-guided minimally invasive treatments (MITs), including ethanol ablation (EA) and thermal ablations, represent an alternative to surgery or even the first-line therapy for benign and locally symptomatic thyroid nodules (TNs) [1–3]. EA is recognized as the best therapeutic approach for relapsing and symptomatic, benign purely cystic thyroid nodules (CTNs) (i.e., cystic portion > 90%) or predominantly cystic thyroid nodules (PCTNs) (i.e., cystic portion > 50%) [1–3]. EA is associated with high volume reduction ratio (VRR) that range from 80% to 100% and from 65% to 85.4% in purely CTNs and PCTNs, respectively [4, 5]. Of note, the high efficacy of EA in reducing thyroid nodule volume persists over time [4, 5]. Several studies have assessed the efficacy of EA for CTNs, especially in terms of VRR and less concerning the amelioration of local symptomatology [5]. Moreover, EA can be considered a safe procedure, as its complications are generally transient and well manageable.

The standardization of EA procedure for CTNs represents a global goal, but several major technical issues remain to be addressed. One unresolved question is how to perform EA when the cyst that is too viscous to be aspirated. The proposed strategies include using larger or open-window intervention needles, with or without repetitive room-temperature saline irrigation,6–10 to achieve liquid aspiration; alternatively, the injection of ethanol prior to aspiration could reduce the viscosity of CTNs[11–15] (a phenomenon called by someone the Saint Januarius effect), thereby favouring the aspiration of cystic content. In this scenario, it is not known whether the contrariwise procedure of EA (contrEA) for CTNs (ethanol injection before aspiration) can achieve the high efficacy level as the classical EA procedure (classEA), which provides the aspiration of almost all the liquid content before ethanol injection. The contrEA is a rescue procedure for a distinct pathological entity, rather than an alternative for the general population, as it is determined by the physical properties of the cyst fluid. Moreover, there is a heated debate on what is the optimal starting dose of ethanol for each session of EA to treat CTNs [14, 16], and it is unclear if similar ethanol doses may determine equal VRR and therapeutic success ratio (TSR) [1–3, 16].

However, in clinical practice other factors seem to influence the effectiveness of EA in shrinking thyroid nodules [i.e., the thyroid nodule composition, the baseline nodule volume, the aspirated liquid volume, the retention or the aspiration of ethanol after evacuating the internal cystic content, the nodule vascularity, the presence of septations (multilocular nodules), and the duration of follow-up] [1–3, 16].

Therefore, the purposes of this retrospective cohort study on single-session EA for CTNs with one year follow-up were (1) to evaluate the VRR, the TSR, and the local symptom improvement among nodules undergoing the classEA or the contrEA procedure and (2) to evaluate the influence of total ethanol amount (mL) on achievement of efficacy outcomes in nodules undergoing the classEA or the contrEA procedure.

## Methods

### Patients with benign and symptomatic CTNs undergoing EA

From June 1, 2021, to September 30, 2025, consecutive patients treated with EA for cytologically benign and symptomatic CTNs were recruited from the endocrine outpatient clinics of our University Hospital. In our institution EA was performed as the first line therapy of symptomatic CTNs which regrow after one aspiration attempt. For PCTNs, benignity was obtained from the cytological analysis of the solid component. Patients were eligible for inclusion if they were euthyroid at baseline, they had negative serum calcitonin and had planned EA for the purpose of volume reduction of thyroid nodules. Exclusion criteria included pregnancy, incomplete follow-up data (less than one year follow-up), and more than one session of classEA per nodule. Patients were excluded if they did not give informed consent to participate in the study. Anthropometric and sociodemographic parameters, and details on EA complications, were obtained by chart review. The thyroid nodule volume was determined by US using the half cube formula, as recommended by American Thyroid Association (ATA) guidelines [17].

EA was performed by the same experienced operator (L.S., with 7 years of clinical experience in performing EA), and it consisted of one to two punctures for sessions, through a needle of 23 Gauge. During US guidance, a lateral approach was used, and local anesthesia was not adopted. Anticoagulation therapy was discontinued ten days before EA, while antiplatelet therapy was not discontinued. The internal fluid of CTNs was aspirated as much as possible by using a syringe of 10 mL or 20 mL or 50mL. 96% ethanol was injected before (contrEA) or after cystic content aspiration (classEA) in the amount of 5–30% of the initial nodule volume (≤ 10mL for each session), without early ethanol reaspiration. The exact volume of ethanol injected was decided during the procedure, based on the following features: the aspirated liquid volume, the nodule location, the thickness of the cystic wall, and the patient tolerance. ContrEA was performed when the aspiration of liquid content was initially not possible after two attempts, one with a needle of 23 Gauge and the second with a needle of 22 Gauge. ContrEA involved that ethanol was left inside the nodule for one week, which consequently allowed the successful aspiration (Saint Januarius effect). After the successful aspiration of the cystic content, no additional doses of ethanol were injected.

Patients were discharged after a 30-min observation period.

Patient follow-up, along with thyroid nodule US, was carried out one, three, six and twelve months after EA, and then annually, according to the international consensus statement [1]. Any additional sessions of EA were carried out after two-three months to further reduce the liquid content or in case of regrowth. Regrowth was defined as a more than 50% increase in total volume compared to the previously reported smallest volume on US. The current retrospective study was conducted in accordance with the Declaration of Helsinki, and it was approved by the Institutional Review Board of our university.

### Outcomes

VRR was calculated using the formula: VRR = (preoperative volume-follow-up volume)/preoperative volume × 100%. TSR was reached when VRR was > 50% one year after EA. Symptom score was assessed on a visual analogue scale (VAS) of 0 (no symptoms) to 10 (maximal symptoms) for each scale (neck pain, dysphasia, foreign body sensation, discomfort, cough [1]. Cosmetic score (CS) was determined by the physician on a scale of 1–4: 1, no palpable nodule; 2, no cosmetic problem but palpable nodule; 3, cosmetic problem only on swallowing; and 4, readily detected cosmetic problem [1]. For the current study, we retrieved the VRR, the symptom scores and cosmetic scores of the baseline and the one-year follow-up visit. The best outcomes for each nodule one year after EA were the following: VRR > 75%, VAS overall score of 0 and cosmetic score of 1; when at least one of these last three outcomes was reached, EA was associated with high local efficacy.

### Statistical analysis

Descriptive statistics were used to characterize the demographic and clinical characteristics of all participants in the study sample. The Kolmogorov-Smirnov test was used to analyse the variables distribution. Continuous variables were presented as median and interquartile ranges (IQR) according to sample distribution. Categorical variables were presented as frequencies and percentages. Mann–Whitney Rank Sum test and the Chi-square test were performed to assess the differences of continuous and categorical variables between groups, respectively. We set VRR as the dependent variable and total ethanol amount, type of procedure (classEA = 0 and contrEA = 1), thyroid nodule volume, and monolocular aspect as the independent variables to evaluate their relationship through a multivariate linear regression analysis. The following three primary outcomes (efficacy outcomes) 12 months after EA were explored: therapeutic success, defined as VRR > 75%; resolution of local symptomatology by patients, defined as overall VAS score = 0 or as CS = 1. To identify predictors for each outcome, a two-step regression approach was used. First, an initial probit model for each outcome evaluated the unadjusted effects of the total ethanol amount and the treatment group. Second, a full multivariate probit model was developed to adjust these findings for potential confounders. Each multivariate model included the total ethanol volume (mL) as a predictor, which was adjusted for the nodule baseline volume (mL) and the presence of internal septations at US (mono- or multilocular nodules). P-value lower than 0.05 was considered statistically significant. All statistical analyses were performed using SPSS software (version 27.0, SPSS, Chicago, IL, USA).

### Patient consent and ethics in pubblication

Informed consent was obtained from all subjects involved in the study, after full explanation of the purpose and nature of all procedures used, according to the protocol approved by the Institutional Review Board of University XXX (Campania 2, 2024 0039/i). Authors followed ethics in the publication.

## Results

### Institutional cohort

**Fig. 1:**
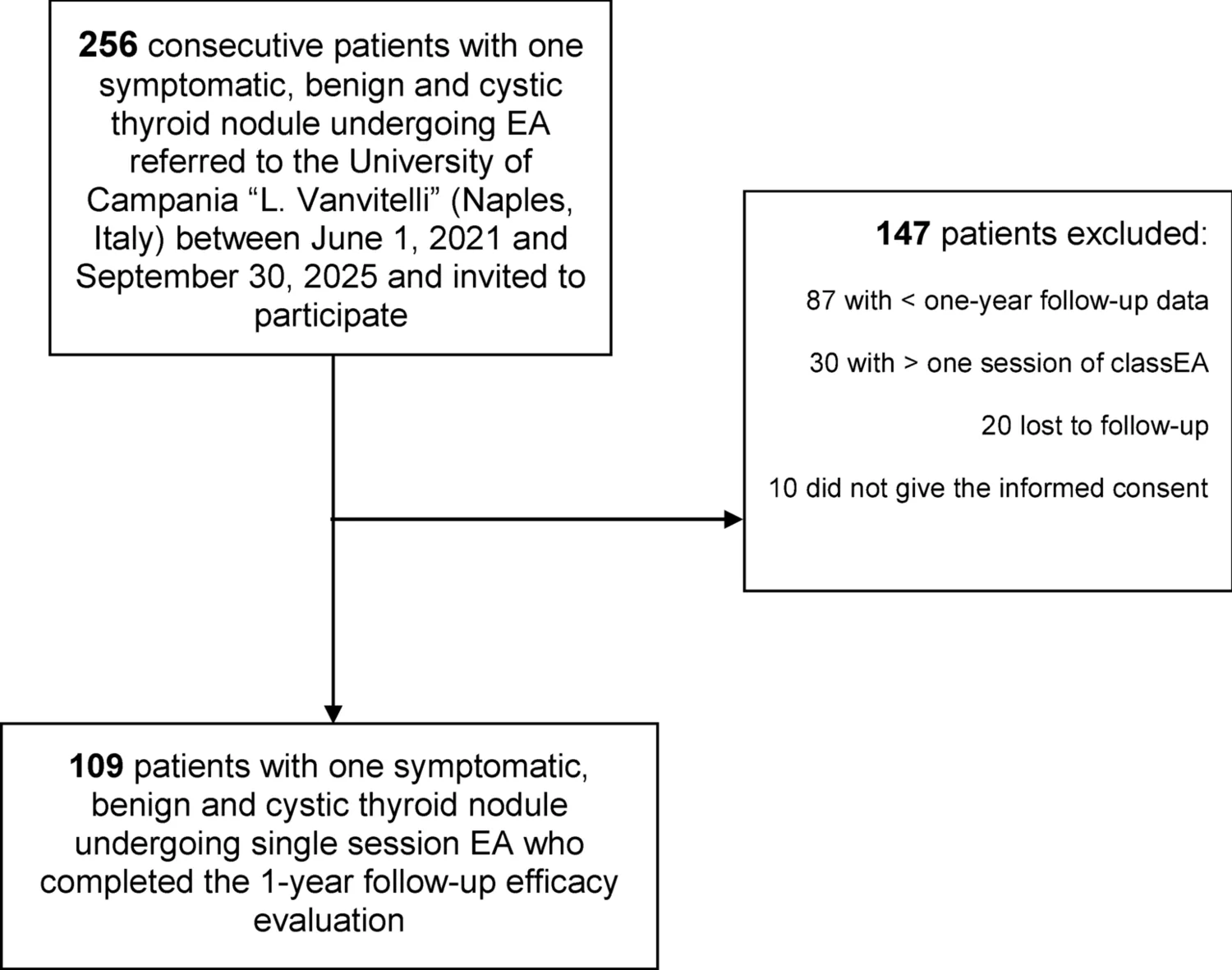
Flow diagram for recruitment and follow-up of patients diagnosed with symptomatic, benign and cystic thyroid nodules, and receiving EA for the purpose of volume reduction. EA, ethanol ablation; classEA, classical ethanol ablation

As shown in Fig. 1, between June 1, 2021, and September 30, 2025, from 256 consecutive patients with one symptomatic, benign cystic thyroid nodule undergoing EA, we excluded 147 patients. We finally included 109 nodules undergoing single-session EA with one year follow-up [median volume 16.2 (7.0-31.7) mL], from 109 patients [65 females and 44 males, median age 49.0 (41.0-60.3) years old]. Table 1 summarizes the clinical and demographic characteristics of the included cohort. Participants were divided into patients undergoing contrEA (*n* = 20) and classEA (*n* = 89), according to the EA procedure. Baseline nodule characteristics, including volume (16.2 mL), maximum diameter (39 mm) and multilocular aspect, were similar between the two groups, with no statistically significant difference; baseline local symptomatology did not statistically differ between the two groups [for contrEA, overall VAS score was 27.0 (24.0-31.5) and for classEA overall VAS score was 26.0 (22.0–30.0), *P* = 0.583; for contrEA, CS was 4.0 (3.0–4.0), and for classEA, CS was 3.0 (3.0–4.0), *P* = 0.448)]. The total amount of injected ethanol did not significantly differ between the two groups (contrEA, median 2.3 vs. classEA, median 2.2, mL, *P* = 0.399). After 6 and 12 months, the classEA group showed a significantly greater VRR compared to contrEA group [83.5% vs. 52.7% (*P* = 0.002) and 93.1% vs. 85.7% (*P* = 0.001), respectively]. TSR was found to be significantly higher in the classEA group than in the contrEA group at six months and 12 months following EA (96.6% vs. 65% at six months, and 100% vs. 65% at 12 months, *P* < 0.001). Twelve months after EA, overall VAS score and CS did not significantly differ between the two groups [for contrEA, VAS 0.0 (0.0–5.0) and for classEA, VAS 0.0 (0.0–1.0), *P* = 0.248; for contrEA, CS 1.0 (1.0–1.0) and for classEA CS 1.0 (1.0–1.0), *P* = 0.405]. The rate of complications was similar between the groups (30.0% in the contrEA group vs. 21% in the classEA group, *P* = 0.754), with a low incidence of major complications (1.0% in the study cohort). No nodules had regrowth. Table 2 shows that classEA and monolocular aspect, but not total ethanol or thyroid nodule volume, are significantly associated with VRR 12 months after EA (β coefficient = -17.04 and *P* < 0.001, β coefficient = -11.92 and P 0.004, respectively).

**Table 1:** Clinical and demographic characteristics of the included patients undergoing EA for benign and cystic thyroid nodules (CTNs) divided according to ethanol injection before [contrariwise EA (contrEA)] or after [classical EA (classEA)] cystic content aspiration

Variables	Study cohort (*n* = 109)	ContrEA (*n* = 20)	ClassEA (*n* = 89)	*P*-value
Age, years	49.0 (41.0-60.3)	52.0 (44.0–56.0)	49.0 (41.0–62.0)	0.541
*Gender*,* n (%)*				0.194
Female	65 (59.6)	15 (75.0)	50 (56.2)	
Male	44 (40.4)	5 (25.0)	39 (43.8)	
*Nodules with septations*,* n (%)*				
Multilocular aspect at US	21 (19.3)	5 (25.0)	16 (17.9)	0.685
*Baseline nodule size*				
Volume, mL	16.2 (7.0-31.7)	16.2 (15.0–21.0)	14.8 (7.0–35.0)	0.501
Dmax, mm	39 (29.8–49.0)	41.0 (39.0-43.3)	39.0 (29.0–49.0)	0.953
Total ethanol amount, mL	2.2 (1.2–3.6)	2.3 (2.0-3.9)	2.2 (1.2–3.2)	0.399
*Nodule size*,* after 6 months*				
Volume, mL	1.9 (1.2-8.0)	8.6 (0.9–9.5)	1.9 (1.2–5.5)	0.123
VRR, %	81.7 (66.7–93.2)	52.7 (39.4–88.0)	83.5 (72.5–93.2)	0.002
*Nodule size*,* after 12 months*				
Volume, mL	1.0 (0.3–2.9)	2.6 (0.6–9.5)	1.0 (0.3–2.8)	0.066
VRR, %	88.9 (77.2–97.6)	85.7 (39.4–88.0)	93.1 (80.4–98.2)	0.001
*Number of nodules achieving TSR after 12 months*				
T6	99 (90.8)	13 (65.0)	86 (96.6)	< 0.001
T12	102 (93.6)	13 (65.0)	89 (100)	< 0.001
*VAS*,* overall score*				
VAS score T0	26.0 (22.0–30.0)	27.0 (24.0-31.5)	26.0 (22.0–30.0)	0.583
VAS score T6	5.0 (0.0–7.0)	5.0 (0.0–16.0)	5.0 (0.0-7-0)	0.737
VAS score T12	0.0 (0.0-1.5)	0.0 (0.0–5.0)	0.0 (0.0–1.0)	0.248
*Cosmetic score*				
Cosmetic score T0	4.0 (3.0–4.0)	4.0 (3.0–4.0)	3.0 (3.0–4.0)	0.448
Cosmetic score T6	1.0 (1.0–2.0)	1.0 (1.0–2.0)	1.0 (1.0-1.3)	0.090
Cosmetic score T12	1.0 (1.0–1.0)	1.0 (1.0–1.0)	1.0 (1.0–1.0)	0.405
*Complications*,* n (%)*	27 (24.8)	6 (30.0)	21 (23.6)	0.754
Minor Complications	26 (23.85)	6 (30.0)	20 (22.47)	0.672
Major complications	1 (0.9)	0 (0.0)	1 (1.12)	0.411

EA, ethanol ablation; US, ultrasound; VRR, volume reduction ratio; TSR, therapeutic success ratio (VRR > 50%); VAS, visual analogue scale; CS, cosmetic score; T0, baseline (before EA); T6, 6 months after EA; T12, 12 months after EA

Continuous variables are expressed in median and interquartile range (IQR), and categorical variables with numbers and percentages. Minor complications: 15 cases of transient burning sense, 7 cases of local pain, 2 cases of neck hematoma, 2 cases of set of symptoms including dizziness/drunkenness and/or flushing and/or increased heartbeat and/or nausea and/or headache. Major complications: 1 case of transient dysphonia

**Table 2:** Linear regression model for predictors of achieving volume reduction ratio (VRR) 12 months after EA in the study cohort (*n* = 109)

VRR	β coefficient	*P*-value
Total ethanol amount, mL	0.334	0.694
Type of EA	-17.04	< 0.001
Thyroid nodule volume, mL	0.043	0.637
Monolocular aspect	-11.92	0.004

EA, ethanol ablation

Type of EA: Classic (Class)EA = 0, contrariwise (Contr)EA = 1

### VRR > 75%

**Fig. 2:**
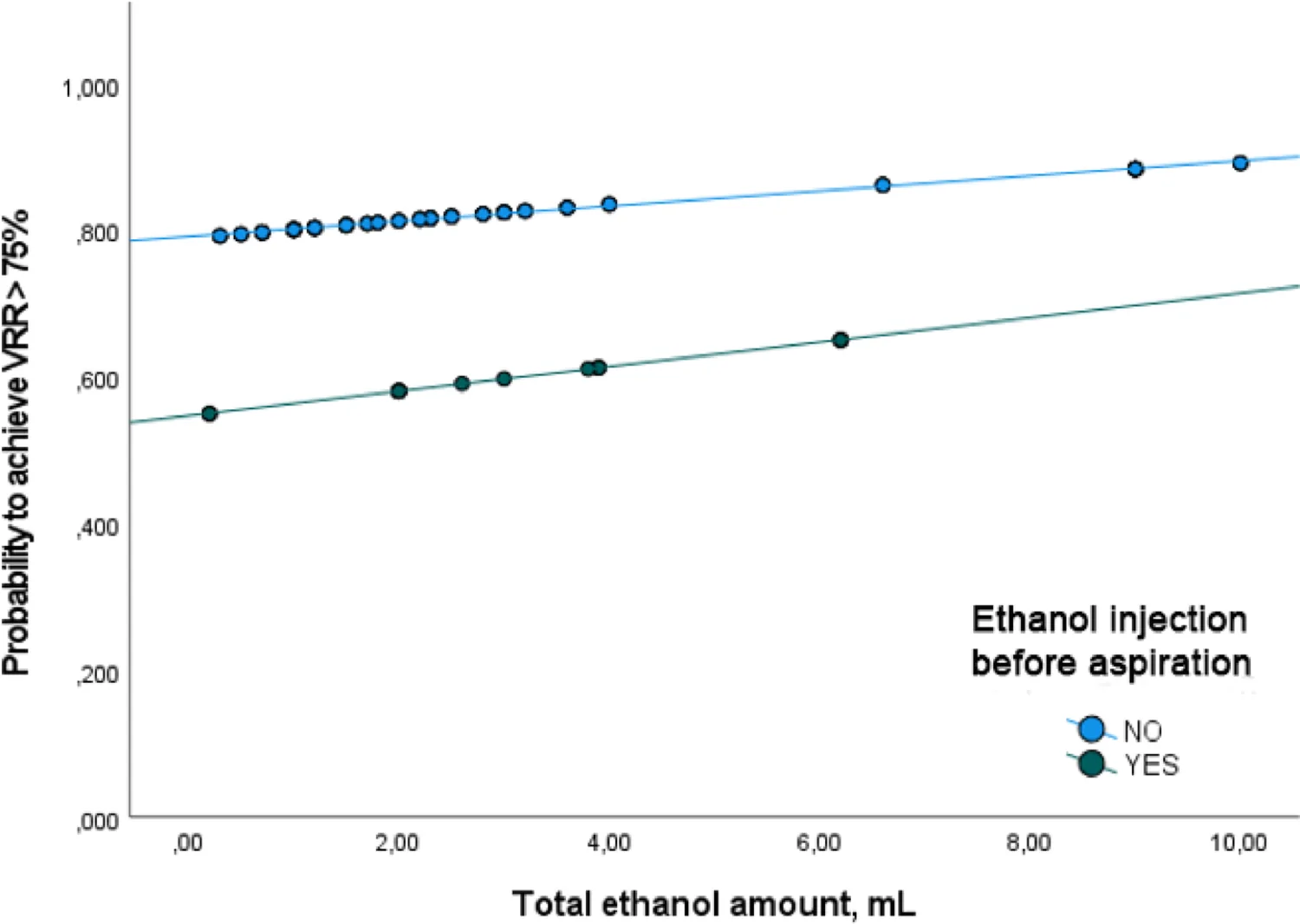
Probit regression model analysis in the cohort undergoing ethanol ablation (EA) of benign and cystic thyroid nodules (CTNs) for ethanol amount in achieving volume reduction ratio (VRR) > 75%, based on EA procedure [i.e., ethanol injection before (contrariwise EA) or after (classical EA) cystic content aspiration]. Total ethanol amount, β = 0.043, *P* = 0.474; type of EA procedure, β = 0.684, *P* = 0.034

The initial probit regression model showed no statistically significant relationship between the amount of injected ethanol and the outcome VRR > 75% (β = 0.043, *P* = 0.474). This regression model also revealed that a statistically significant difference existed between the two treatment groups, with the classEA group showing a higher probability of success (β = 0.684, *P* = 0.034) (Fig. 2). In the full multivariate probit regression model, the effect of the type of procedure (contrEA or classEA) was no longer statistically significant (β = 0.615, *P* = 0.077), while monolocular aspect of thyroid nodules was identified as the only significant predictor of success [β = 0.820, Odds ratio (OR) 2.271 (1.143; 4.513), *P* = 0.019]. The total ethanol amount remained non-significant (β = 0.052, *P* = 0.580). Given the absence of a significant association between predictor and outcome, it was not allowed to estimate a specific ethanol dose to administer during the EA procedure to achieve a given probability of success for this outcome (Table 3).

**Table 3:** Multivariable probit regression model for predictors of achieving volume reduction ratio (VRR) > 75% in the study cohort (*n* = 109)

VRR	β coefficient (95% CI)	OR (95% CI)	*P*-value
Total ethanol amount, mL	0.052 (-0.133; 0.238)	1.054 (0.875; 1.268)	0.580
Type of EA	0.615 (-0.067; 1.298)	1.851 (0.935; 3.663)	0.077
Thyroid nodule volume, mL	0.006 (-0.016; 0.028)	1.006 (0.984; 1.028)	0.596
Monolocular aspect	0.820 (0.133; 1.507)	2.271 (1.143; 4.513)	0.019

EA, ethanol ablation; CI, Confidence interval; OR, odds ratio

Type of EA: Classic (Class)EA = 0, contrariwise (Contr)EA = 1

### Overall VAS score = 0

The initial probit regression model showed that there was no statistically significant relationship between the amount of injected ethanol, the type of EA procedure (contrEA or classEA) and the outcome overall VAS score = 0 (total ethanol amount, β = -0.028, *P* = 0.574; type of EA procedure, β = 0.288, *P* = 0.365) (Supplementary Fig. 1). The full multivariate probit regression model revealed that monolocular aspect of thyroid nodules was statistically and significantly associated with a probability to achieve the outcome overall VAS score = 0 [β = 1.459, OR 4.303 (2.157; 8.583), *P* < 0.001]. The remaining variables were not statistically significant (Table 4).

**Table 4:** Multivariable probit regression model for predictors of achieving overall visual analogue scale (VAS) score = 0 in the study cohort (*n* = 109)

VAS score	β coefficient (95% CI)	OR (95% CI)	*P*-value
Total ethanol amount, mL	0.081(-0.255; 0.093)	1.085 (0.912; 1.291)	0.359
Type of EA	0.345 (-1.041; 0.350)	1.412 (0.704; 2.832)	0.331
Thyroid nodule volume, mL	-0.010 (-0.007; 0.270)	0.990 (0.974; 1.007)	0.243
Monolocular aspect	1.459 (-2.150; -0.769)	4.303 (2.157; 8.583)	< 0.001

EA, ethanol ablation; CI, Confidence interval; OR, odds ratio

Type of EA: Classic (Class)EA = 0, contrariwise (Contr)EA = 1

### CS = 1

The initial probit model found that neither the total amount of ethanol nor the type of procedure (contrEA or classEA) was significantly associated with CS = 1 (total ethanol amount: β = 0.004, *P* = 0.946; type of procedure: β = 0.213, *P* = 0.554) (Supplementary Fig. 2). In the full multivariate probit regression model, monolocular aspect of thyroid nodules emerged as the only significant predictor of CS = 1 [β = 1.689, OR 5.416 (2.508; 11.697), *P* < 0.001]. The remaining variables were not statistically significant (Supplementary Table 1).

## Discussion

Technical aspects of EA for benign and symptomatic CTNs should be internationally standardized [1–3, 16]. Several crucial technical issues need to be resolved before this objective can be realized, including the appropriate approach with EA when aspiration is not feasible and the definition of the optimal ethanol amount to be used for each EA session [1–3, 11, 12, 16]. Extracting the liquid content of CTNs before ethanol injection is the technical mainstay for a classEA procedure associated with high efficacy [11, 12]. In clinical practice, in some cases aspiration of CTNs cannot be pursued because of the high viscosity of liquid content [1–3, 11, 12]. When this happens, it is unclear whether and how to perform an efficacious EA procedure. However, we should generally adopt the best EA procedure in terms of simplicity of execution and efficacy; and, although EA is deemed a safe MIT for the treatment of CTNs[18], we should follow the principle of the minimal therapeutic dose of ethanol to obtain the best outcome.

The study addresses a valid clinical difficulty: managing cysts with highly viscous content. Over a period of four years, we retrospectively analyzed the impact of the EA procedure (with or without the Saint Januarius effect) and the injected ethanol dose on efficacy of single-session PEI for 109 benign and symptomatic CTNs at one year follow-up. The Saint Januarius effect is so named because it refers to the miracle of the liquefaction of the blood of St. Januarius (bishop, martyr, and patron saint of Naples, Italy) (https://www.catholicnewsagency.com). The contrEA group represents a “difficult-to-treat” cohort (characterized by a viscous fluid that failed initial aspiration), whereas the classEA group represents standard, less viscous cysts.

First, we found that in 18% of nodules cystic content aspiration was not possible; in these cases, the contrEA was performed. Then, we demonstrated that VRR after 6 and 12 months was higher in classEA (83.5% and 93.1%, respectively), compared with contrEA (52.7% and 85.7%, respectively), with size (~ 16.0 mL, 39 mm) and local symptomatology (overall VAS score 26.0 and CS 4.0) overlapping at baseline. Thus, in terms of VRR, the largest difference between the two EA procedures was seen after 6 months, while after 12 months there was a significant but minor difference between the two EA procedures. Although EA for CTNs is generally regarded as a MIT associated with high and rapid results[1–3, 11, 12], we could admit that contrEA, compared with classEA, produces VRR more slowly. This is probably because in the contrEA procedure, the aspiration of liquid content, after leaving ethanol for only one week inside the nodule, could delay the effect of nodule impairment. In the classEA procedure, leaving ethanol inside the nodule after aspiration of the cystic content may account for the faster VRR, compared with contrEA. The lower efficacy speed in the contrEA group could also be likely due to the nature of the cyst itself rather than the timing of the injection.

When we explored baseline features of our cohort potentially influencing the VRR after 12 months (i.e., the injected ethanol amount, the type of EA procedure, the thyroid nodule volume, and the monolocular aspect of nodules at US), we found that the classEA procedure and the monolocular aspect of thyroid nodules are associated with VRR increase. However, at the six- and twelve-month follow-up, there was not a difference between the two EA procedures groups in terms of quality and speed of amelioration of local symptomatology (assessed with the overall VAS score and the CS).

Moreover, we attempted to explore the potential of the injected ethanol amount to predict three high efficacy outcomes (i.e., VRR > 75%, overall VAS score = 0 and CS = 1) twelve months after single session EA of CTNs. We showed that the amount of injected ethanol could not predict these three outcomes through the initial probit regression model. In other words, in our cohort this finding could highlight the principle that greater or lesser amounts of ethanol may similarly achieve excellent efficacy outcomes. In addition, the full multivariate probit regression model proved that the achievement of the three high efficacy outcomes could be significantly predicted only by the monolocular aspect of thyroid nodules, without the contribution of ethanol amount, EA procedure, and thyroid nodule volume. Therefore, within our cohort, the monolocular thyroid nodules demonstrated a 2–5 fold greater reponse compared to multilocular thyroid nodules and drive the high efficacy results, regardless of ethanol amount, EA procedure, and thyroid nodule volume. In clinical practice, in CTNs the presence of internal septations could limit the total aspiration of liquid content and the spread of ethanol at the cystic wall, potentially reducing the chemical damage on the nodule [1–3, 11, 12]. Finally, although in the initial probit regression model for VRR > 75% the classEA procedure, compared with the contrEA, showed a higher probability of success, in the full multivariate probit regression model, the effect of the type of procedure (contrEA or classEA) was no longer statistically significant. The latter finding could be explained by the fact that an absolute number of monolocular thyroid nodules belonged to the classEA group.

Our study does have some limitations. First, we did not explore the nodule vascularity or the thyroid nodule composition, as potential factors influencing the ethanol doses and the efficacy of PEI [1–3, 19]. Second, follow-up time could be considered short (one year) [19]. Third, our findings refer to the aspiration of cystic content executed with 22–23 Gauge needles. We think that thin needles can be effective devices for aspiration of CTNs and they are associated with negligible pain for patients when local anesthesia is not adopted. This also aligns with EA regarded as a MIT that is easy to perform (i.e., no local anesthesia, thin needles). In other working groups [6, 8–10], typically after local anesthesia, if the cysts contain high-viscosity fluid, this was aspirated using a larger-bore needle (16 to 18 Gauge). However, the superiority of larger over thinner needles in aspiration of CTNs has never been proved. Fourth, in our contrEA procedure, after aspirating almost all the cystic content thanks to the Saint Januarius effect, no further doses of ethanol were injected. In other studies, when the cystic content was impossible to aspirate or it was just minimally aspirated, additional doses of ethanol were injected inside the nodule after aspiration [11–13, 15]. This EA procedure was named “two-stage EA” since a repeated EA was performed 1–3 months after the initial ethanol injection for the liquefaction of viscous contents [15]. Conversely, in the current study, in the contrEA group, simple aspiration allowed us to obtain high efficacy outcomes without the need to inject additional ethanol doses. Fifth, the efficacy outcomes are likely to be overestimated, since we excluded nodules undergoing more than one session of EA. Sixth, the small number of nodules undergoing the contrEA and the relatively small doses of ethanol (≤ 10 mL) could limit the power of results of the regression analyses.

A major strength of this study consists in addressing technical issues poorly explored in literature, namely the efficacy of EA when liquid content cannot be initially aspirated and the role of the ethanol amount in the treatment efficacy of EA for CTNs [1–3, 16].

## Conclusions

Based on the results of this study, performing EA with or without the Saint Januarius effect allows us to achieve high efficacy outcomes (i.e., VRR > 75%, disappearance of local symptomatology expressed as overall VAS score = 0 and CS = 1) at the one-year follow-up. Monolocular aspect of thyroid nodules seems to be a factor that positively influences the efficacy of EA, regardless of the EA procedure (with or without the Saint Januarius effect).

## Supplementary Information

Below is the link to the electronic supplementary material.

Supplementary Material 1

Supplementary Material 2

Supplementary Material 3

Supplementary Material 4

Supplementary Material 5

## Data Availability

The datasets used and/or analyzed during the current study are available from the corresponding author upon reasonable request.
