# Supplementary material for: Ablation of cystic thyroid nodules with or without the injection of ethanol prior to aspiration: a one-year follow-up study
Source: Updates Surg. 2026 May 5;78(6):2621–8. doi: 10.1007/s13304-026-02607-9 (PMC13736405; doi:10.1007/s13304-026-02607-9)
Supplement: Supplementary file 1 [file 13304_2026_2607_MOESM1_ESM.docx]

Naples, 20/12/2025

Professor Ugo Boggi, MD, FEBS, University of Pisa, Italy
and Professor Fulvio Calise, MD, Cardarelli Hospital, Naples, Italy

Editors in Chief of *Updates in Surgery*

Dear Professors Ugo Boggi and Fulvio Calise

We are pleased to submit our original manuscript entitled “**Ethanol ablation of cystic thyroid nodules with or without the Saint Januarius effect: a one-year follow-up study”** for consideration for publication in *Updates in Surgery.*

This study aimed to evaluate volume reduction rates (VRRs) and local symptom improvement among cystic thyroid nodules (CTNs) according to the ethanol ablation (EA) procedure [with, contrariwise EA (contrEA), or without, classical (classEA), the Saint Januarius effect] and injected ethanol amounts. To this purpose, we retrospectively reviewed consecutive patients treated with single-session EA for cytologically benign and symptomatic CTNs over a period of four years at our University hospital, including a total of 109 nodules [contrEA (n = 20) and classEA (n = 89)]. We found that EA, with or without the Saint Januarius effect, is effective to achieve high efficay outcomes (i.e., VRR>75%, overall VAS score=0, CS=1) at one year follow-up and the the efficacy seems to be positively influenced by monolocular aspect of thyroid nodules, regardless of the ethanol injected, EA procedure, thyroid nodule volume.

The manuscript has not been published or submitted elsewhere, and all authors have read and approved the final version. No funding was received for this study, and all potential conflicts of interest are fully disclosed within the manuscript. We request that the article should be considered for publication as an Open Access original article.

We thank you for considering our work for publication in *Updates in Surgery.* We look forward to your response.

Sincerely,
Lorenzo Scappaticcio, MD PhD

Department of Advanced Medical and Surgical Sciences, University of Campania "Luigi Vanvitelli", 80138 Naples, Italy; [lorenzo.scappaticcio@unicampania.it](mailto:lorenzo.scappaticcio@unicampania.it); +39-0815665032
