# Supplementary material for: Ablation of cystic thyroid nodules with or without the injection of ethanol prior to aspiration: a one-year follow-up study
Source: Updates Surg. 2026 May 5;78(6):2621–8. doi: 10.1007/s13304-026-02607-9 (PMC13736405; doi:10.1007/s13304-026-02607-9)
Supplement: Supplementary file 2 [file 13304_2026_2607_MOESM2_ESM.docx]

**Figure captions**

**Figure 1**. Flow diagram for recruitment and follow-up of patients diagnosed with symptomatic, benign and cystic thyroid nodules, and receiving EA for the purpose of volume reduction.

EA, ethanol ablation; classEA, classical ethanol ablation

**Figure 2.** Probit regression model analysis in the cohort undergoing ethanol ablation (EA) of benign and cystic thyroid nodules (CTNs) for ethanol amount in achieving volume reduction ratio (VRR) > 75%, based on EA procedure [i.e., ethanol injection before (contrariwise EA) or after (classical EA) cystic content aspiration]. Total ethanol amount, β = 0.043, P = 0.474; type of EA procedure, β = 0.684, P = 0.034.
