# Supplementary material for: Ablation of cystic thyroid nodules with or without the injection of ethanol prior to aspiration: a one-year follow-up study
Source: Updates Surg. 2026 May 5;78(6):2621–8. doi: 10.1007/s13304-026-02607-9 (PMC13736405; doi:10.1007/s13304-026-02607-9)
Supplement: Supplementary file 3 [file 13304_2026_2607_MOESM3_ESM.docx]

**Declaration of competing interest**

The authors declare no conflicts of interest.
