# Supplementary material for: Ablation of cystic thyroid nodules with or without the injection of ethanol prior to aspiration: a one-year follow-up study
Source: Updates Surg. 2026 May 5;78(6):2621–8. doi: 10.1007/s13304-026-02607-9 (PMC13736405; doi:10.1007/s13304-026-02607-9)
Supplement: Supplementary file 4 [file 13304_2026_2607_MOESM4_ESM.docx]

**Ablation of cystic thyroid nodules with or without the injection of ethanol prior to aspiration: a one-year follow-up study**

**Supplementary material**

**Supplementary figure 1:** Probit regression model analysis relative to ethanol amount to achieve overall visual analogue scale (VAS) = 0, based on EA procedure; pag.2

**Supplementary figure 2:** Probit regression model analysis relative to ethanol amount to achieve cosmetic score (CS) = 1, based on EA procedure; pag.3

**Supplementary table 1:** Multivariable probit regression model relative to cosmetic score (CS), pag.4

**Supplementary figure 1.** Probit regression model analysis in the cohort undergoing ethanol ablation (EA) of benign and cystic thyroid nodules (CTNs) for ethanol amount in achieving overall visual analogue scale (VAS) score = 0, based on EA procedure [i.e., ethanol injection before (contrariwise EA) or after (classical EA) cystic content aspiration]. Total ethanol amount, β = -0.028, P = 0.574; type of EA procedure, β = 0.288, P = 0.365.

**Supplementary figure 2.** Probit regression model analysis in the cohort undergoing ethanol ablation (EA) of benign and symptomatic cystic thyroid nodules (CTNs) for ethanol amount in achieving cosmetic score (CS) = 1, based on on EA procedure [i.e., ethanol injection before (contrariwise EA) or after (classical EA) cystic content aspiration]. Total ethanol amount: β = 0.004, P = 0.946; type of procedure: β = 0.213, P =0.554

**Supplementary table 1**. Multivariable probit regression model for predictors of achieving cosmetic score (CS) = 1 in the study cohort (n = 109)

| **Cosmetic score** | **β coefficient ( 95% CI)** | **OR (95% CI)** | **P value** |
| --- | --- | --- | --- |
| Total ethanol amount, mL | 0.068 (-0.165; 0.301) | 1.071 (0.848; 1.352) | 0.567 |
| Type of EA | 0.270 (-0.540; 1.080) | 1.310 (0.583; 2.945) | 0.514 |
| Thyroid nodule volume, mL | 0.008 (-0.024; 0.040) | 1.008 (0.976; 1.041) | 0.628 |
| Monolocular aspect | 1.689 (0.919; 2.459) | 5.416 (2.508; 11.697) | <0.001 |

Abbreviation: EA, ethanol ablation; CI, Confidence interval; OR, odds ratio.

Type of EA: Classic (Class)EA = 0, contrariwise (Contr)EA = 1
